# Functional Disconnection and Compensation in Mild Cognitive Impairment: Evidence from DLPFC Connectivity Using Resting-State fMRI

**DOI:** 10.1371/journal.pone.0022153

**Published:** 2011-07-21

**Authors:** Peipeng Liang, Zhiqun Wang, Yanhui Yang, Xiuqin Jia, Kuncheng Li

**Affiliations:** 1 Xuanwu Hospital, Capital Medical University, Beijing, China; 2 The International WIC Institute, Beijing University of Technology, Beijing, China; 3 Beijing Municipal Lab of Brain Informatics, Beijing, China; Beijing Normal University, China

## Abstract

The known regional abnormality of the dorsolateral prefrontal cortex (DLPFC) and its role in various neural circuits in mild cognitive impairment (MCI) has given prominence to its importance in studies on the disconnection associated with MCI. The purpose of the current study was to examine the DLPFC functional connectivity patterns during rest in MCI patients and the impact of regional grey matter (GM) atrophy on the functional results. Structural and functional MRI data were collected from 14 MCI patients and 14 age, gender-matched healthy controls. We found that both the bilateral DLPFC showed reduced functional connectivity with the inferior parietal lobule (IPL), superior/medial frontal gyrus and sub-cortical regions (e.g., thalamus, putamen) in MCI patients when compared with healthy controls. Moreover, the DLPFC connectivity with the IPL and thalamus significantly correlated with the cognitive performance of patients as measured by mini-mental state examination (MMSE), clock drawing test (CDT), and California verbal learning test (CVLT) scores. When taking GM atrophy as covariates, these results were approximately consistent with those without correction, although there may be a decrease in the statistical power. These results suggest that the DLPFC disconnections may be the substrates of cognitive impairments in MCI patients. In addition, we also found enhanced functional connectivity between the left DLPFC and the right prefrontal cortex in MCI patients. This is consistent with previous findings of MCI-related increased activation during cognitive tasks, and may represent a compensatory mechanism in MCI patients. Together, the present study demonstrated the coexistence of functional disconnection and compensation in MCI patients using DLPFC functional connectivity analysis, and thus might provide insights into biological mechanism of the disease.

## Introduction

Alzheimer's disease (AD) is a progressive neurodegenerative disorder, which is characterized by global cognitive decline, including progressive loss of memory, reasoning and language. There has been much anatomical and functional evidence pointing toward AD as a kind of disconnection syndrome [1]–[3]. Some studies have shown disruption of a distributed network, especially the linkages between the hippocampus, the prefrontal cortex and other brain areas [1], [4], [5]. Moreover, a number of studies have reported the compensatory mechanism in AD, as indicated by the increased functional connectivity within the prefrontal lobe or between prefrontal regions and other brain regions [4], [6], [7]. Mild cognitive impairment (MCI) refers to a transitional stage between normal aging and early AD [8]. MCI has a high probability of evolving toward AD at a rate of 10–15% per year [9]. It is thus of theoretical and practical significance to examine if MCI show similar effects of functional disconnection and compensation with AD.

The study of resting state brain function is especially applicable to the study of patients because of the practical advantages of resting-state fMRI in terms of ease of clinical application. Functional connectivity during rest, measured by correlation in low frequency fluctuations (LFF) (<0.1 Hz) of the blood oxygen level-dependent (BOLD) signal, has been used to characterize intrinsic architecture of large-scale brain systems, including motor [10], visual [11], auditory [12], language [13], attention [14] and default mode network (DMN) [15], [16] systems.

Several resting state fMRI studies have demonstrated the abnormalities of functional integrity in MCI patients [17]–[19]. For example, based on posterior cingulate cortex (PCC) functional connectivity analysis, Bai et al. [17] indicated regions of decreased functional connectivity were identified in aMCI patients, most notably in temporal cortex (BA 21, 37, 39), whilst significantly increased functional connectivity was mainly in frontal cortex (BA 9, 11, 47), as compared to healthy controls. Using independent component analysis (ICA) approach, Qi et al. [18] compared the default mode network (DMN) activity between MCI patients and healthy elderly, and showed that MCI patients exhibited decreased functional activity in the DMN regions, including the bilateral precuneus/PCC, right inferior parietal lobule, and left fusiform gyrus, while increased activity in the left prefrontal cortex (BA 8) and other parietal, temporal regions, as compared to the healthy elderly. Most of these studies showed that functional disconnection and compensation coexisted in MCI patients, however, they mainly focused on the DMN, i.e., the task-negative network.

The dorsolateral prefrontal cortex (DLPFC), different from DMN regions (e.g., PCC/precuneus, medial prefrontal cortex, etc.), is an important region of task-positive. Numerious evidences have demonstrated that DLPFC plays crucial roles in various attention-demanded cognitive tasks such as working memory and executive function [20], episodic memory [21], attention [22], decision making [23], reasoning [24] and problem solving [25], and so on. Functional and structural studies also indicated that DLPFC is connected to a variety of brain areas, including the thalamus, basal ganglia, the orbitofrontal cortex and primary and secondary association areas of neocortex, including posterior temporal, parietal, and occipital areas [26], [27]. Although abnormal activities of the DLPFC as well as functional disconnection involving DLPFC have been observed in MCI and AD [18], [28]–[30], the precise and detailed connection patterns of the DLPFC in MCI are still unclear. Considering its local dysfunction and roles in various neural circuits relevant to the physiological mechanisms of cognitive impairment, the functional connectivity of the DLPFC in MCI should be especially emphasized. However, to our knowledge so far, no study has examined the DLPFC functional connectivity (DLPFC-FC) in MCI patients.

Using resting state functional connectivity and graph theory analysis, Dosenbach et al. [27] proposed that there are two commanders in the brain: fronto-parietal and cingulo-opercular circuits. The fronto-parietal circuit mainly comprises of premotor cortex (BA 6), DLPFC (BA 46, 47), IPL (BA 40) and ACC (BA 24/32). Two previous resting state fMRI studies observed the similar fronto-parietal network by using probabilistic independent component analysis (PICA) [14] or seed-based correlation analysis [31]. Moreover, in an automatic meta-analysis of 825 neuroimaging articles representing 3402 experiments, Toro et al. [32] obtained a similar co-activation map of fronto-parietal network. The fronto-parietal network in these studies was called as “task-positive” network, which was activated during performance of attention-demanding cognitive tasks. The cingulo-opercular circuit is composed of the medial frontal cortex (BA 6, 8), insula (BA 13), anterior prefrontal cortex (aPFC; BA 11/10), and thalamus. This pathway was recruited in many cognitive tasks, e.g., cognitive control [33], learning [34] and planning [35], etc.

The first goal of the present study is then to investigate the resting-state functional connectivity patterns of the DLPFC in MCI patients. It was hypothesized that the effects of functional disconnection of the two circuits and compensation within lobes coexisted in AD can be detected in its early stage, i.e., MCI, by using DLPFC-FC analysis.

The second goal of this study is to examine the asymmetry of DLPFC-FC in MCI patients. The different role of the bilateral DLPFC is well-known in working memory [36], episodic memory [37] for healthy adults. We thus expect to observe the functional asymmetry of DLPFC-FC in healthy controls, and are interested to test whether the functional asymmetry of DLPFC-FC in MCI patients is altered as compared to healthy controls.

## Materials and Methods

### Subjects

Twenty-eight right-handed subjects (14 MCI patients and 14 healthy elders) participated in this study. The MCI subjects were recruited from patients who had consulted a memory clinic for memory complaints at Xuanwu Hospital, Beijing, China. The criteria for identification and classification of subjects with MCI [9] was: (a) impaired memory performance on a normalized objective verbal memory delayed-recall test; (b) recent history of symptomatic worsening in memory; (c) normal or near-normal performance on global cognitive tests [Mini-Mental State Examination (MMSE) score >24], as well as on activities of daily living scale; (d) global rating of 0.5 on the CDR Scale, with a score of at least 0.5 on the memory domain; (e) absence of dementia. The healthy elderly controls were recruited from the local community by advertisements. The criteria for healthy elderly controls were as follows: (i) matched with MCI patients by the gender, age and education level; (ii) had no cognitive complaints and no positive sign in the neurological exam; (iii) MMSE score of 28 or higher.

The demographic and clinical data were shown in Table 1. The study was approved by the Research Ethics Committee of XuanWu Hospital, Capital Medical University and written informed consent was obtained from each participant.

**Table 1:** Demographics and clinical findings.

	MCI(*N* = 14)	Controls(*N* = 14)	*p* value
Sex, female/male	8/6	8/6	>0.99#
Age, year	69.64±6.88	68.07±7.46	0.583*
Education, year	11.00±3.59	9.14±3.66	0.082*
MMSE^a^	26.64±1.01	28.57±0.65	<0.05*
CVLT(immediate)	7.51±0.85	10.84±0.57	<0.05*
CVLT(short time)	6.76±0.48	11.16±0.54	<0.05*
CVLT(long-time)	5.47±0.76	11.60±0.58	<0.05*
CDT	5.54±0.52	9.65±0.45	<0.05*

MMSE, Mini-Mental State Examination; values are means ± SD. WHO-UCLA CVLT, California verbal learning test; immediate, immediate recall of learning verbal; short-time, short time delayed free recall; long-time, long time delayed free recall; CDT, clock drawing test.

# The p value was obtained using a Pearson x^2^ two-tailed test, with continuity correction for n<5.

*The p value was obtained by a two-sample two-tailed t-test.

### fMRI Recording

MRI data acquisition was performed on a SIEMENS Trio 3-Tesla scanner (Siemens, Erlangen, Germany). Foam padding and headphones were used to limit head motion and reduce scanner noise. The subjects were instructed to hold still, keep their eyes closed and think nothing in particular. Functional images were collected axially by using an echo-planar imaging (EPI) sequence [repetition time (TR)/echo time (TE)/flip angle (FA)/field of view (FOV) = 2000 ms/40 ms/90°/24 cm, resolution = 64×64 matrix, slices = 28, thickness = 4 mm, gap = 1 mm, bandwidth = 2232 Hz/pixel]. The scan lasted for 478 s. 3D T1-weighted magnetization-prepared rapid gradient echo (MPRAGE) sagittal images were collected by using the following parameters: TR/TE/inversion time (TI)/FA = 1900 ms/2.2 ms/900 ms/9°, resolution = 256×256 matrix, slices = 176, thickness = 1 mm.

### Data Preprocessing

Unless otherwise stated, all analyses were conducted using a statistical parametric mapping software package (SPM5, http://www.fil.ion.ucl.ac.uk/spm). The first 10 volumes of the functional images were discarded for the signal equilibrium and participants' adaptation to the scanning noise. The remaining 229 fMRI images were first corrected for within-scan acquisition time differences between slices and then realigned to the first volume to correct for inter-scan head motions. No participant had head motion of more than 1.5 mm maximum displacement in any of the x, y, or z directions and 1.5° of any angular motion throughout the course of scan. The individual structural image was co-registered to the mean functional image after motion correction using a linear transformation. The transformed structural images were then segmented into gray matter (GM), white matter (WM) and cerebrospinal fluid (CSF) by using a unified segmentation algorithm [38]. The motion corrected functional volumes were spatially normalized to the Montreal Neurological Institute (MNI) space and re-sampled to 3 mm isotropic voxels using the normalization parameters estimated during unified segmentation. Subsequently, the functional images were spatially smoothed with a Gaussian kernel of 4×4×4 mm^3^ full width at half maximum (FWHM) to decrease spatial noise. Following this, temporal filtering (0.01 Hz<f<0.08 Hz) was applied to the time series of each voxel to reduce the effect of low-frequency drifts and high-frequency noise [10], [11], [15] using Resting-State fMRI Data Analysis Toolkit (http://resting-fmri.sourceforge.net). To further reduce the effects of confounding factors, we also used a linear regression process to further remove the effects of head motion and other possible sources of artifacts [31]: (1) six motion parameters, (2) whole-brain signal averaged over the entire brain, (3) linear drift.

### Functional Connectivity Analysis

The bilateral DLPFC ROIs were generated using the free software WFU_PickAtlas (http://www.ansir.wfubmc.edu) [39]. In the present study, DLPFC refers to the BA 46, which has been used in previous studies [40], [41]. The BOLD time series of the voxels within each seed region were averaged to generate the reference time series for this seed region. For each subject and each seed region, a correlation map was produced by computing the correlation coefficients between the reference time series and the time series of all brain voxels. Correlation coefficients were converted to z values using Fisher's r-to-z transform to improve the normality [11]. For verification, we also perform a coordinate-based DLPFC-FC and PCC functional connectivity (PCC-FC) analysis (See Supplementary Materials: 1).

Then, the individual z value was entered into a random effect one-sample t-test to determine brain regions showing significant connectivity to the left and right DLPFC within each group under a combined threshold of *P*<0.01 and cluster size > = 405 mm^3^. This yields a corrected threshold of *P*<0.05, determined by Monte Carlo simulation using the AlphaSim program with parameters: FWHM = 4 mm, within the GM mask (http://afni.nimh.nih.gov/pub/dist/doc/manual/AlphaSim.pdf). This procedure produced significant DLPFC-FC t-statistic maps for patients and controls. We then made a mask respectively for the bilateral DLPFC by combining the two DLPFC-FC t-statistic maps (i.e., MCI patients, healthy controls), and then used this mask for analyzing the group differences. Such an inclusion masking procedure can constrain our analyses within regions generating potentially meaningful low frequency oscillations. Voxels survived a combined threshold of *P*<0.01 and cluster size > = 216 mm^3^, which corresponds to a corrected threshold of *P*<0.05 (using the AlphaSim program with parameters: FWHM = 4 mm, with mask), were considered to show the group differences.

The z values were also entered into a random effect paired t-test to examine the DLPFC-FC asymmetry for healthy controls and MCI patients, respectively, and two-sample t-test to identify the DLPFC-FC asymmetry differences between MCI patients and healthy controls. Voxels survived a corrected threshold of *P*<0.05 (using the AlphaSim program with parameters: FWHM = 4 mm, with mask) were considered to show significant difference between the two groups.

### Correlation Analysis of DLPFC-FC and Neuropsychological Measures

In order to explore whether DLPFC-FC varies with the disease progression and cognitive performances in MCI patients, a further correlation analysis between DLPFC-FC and neuropsychological performances was performed. First, averaged z-values of each cluster with the significant group differences were extracted. Then, Pearson's correlative analysis were performed to examine relationships between the z-values and neuropsychological performances (including CVLT: Immediate Recall, CVLT: Short Delayed Recall, CVLT: Long Delayed Recall, CDT and MMSE) in MCI patients using SPSS software (SPSS, Inc., Chicago, IL).

### Controlling for Regional GM Atrophy

Brain atrophy may cause a partial volume effect in functional imaging techniques [42]. Recent researches have demonstrated the potential impact of atrophy on the functional results in previous fMRI studies of AD [43], [44]. To explore the possible effect, a voxel-based-morphometry (VBM) analysis of structural images was performed in this study.

GM intensity maps were obtained by the unified segmentation algorithm [38] as described in the Data Preprocessing section. After spatially smoothed with a Gaussian kernel of 10 mm FWHM, a two-sample t-test was performed on the smoothed GM intensity maps between healthy controls and MCI patients. The statistical threshold was set at *P*<0.001 and cluster size >324 mm^3^, which corresponded to a corrected *P*<0.05 (using the AlphaSim program with parameters: FWHM = 10 mm, within the GM mask).

Furthermore, we re-analyzed the DLPFC-FC results (i.e., two-sample t-tests and correlation analysis) by taking voxel-wise GM volume as covariates [43]. The statistical threshold was also set at a corrected *P*<0.05.

## Results

There were no significant differences between the two groups in gender, age, and years of education, but the MMSE, CVLT and CDT scores were significantly different (*P*<0.05) between the two groups.

### DLPFC-FC: Within-group Results

**Figure 1:**
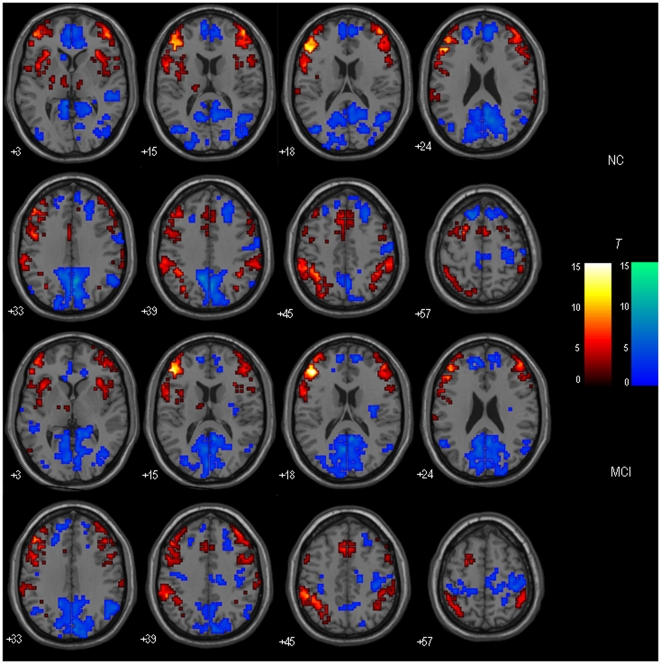
Within-group maps of the left DLPFC-FC in MCI and healthy control groups. The voxels with hot color represent DLPFC positive functional connectivity, and the voxels with cold color represent DLPFC negative functional connectivity. The statistical threshold was set at P<0.01 and cluster size > = 405 mm^3^, which corresponded to a corrected P<0.05. Left is the left.

**Figure 2:**
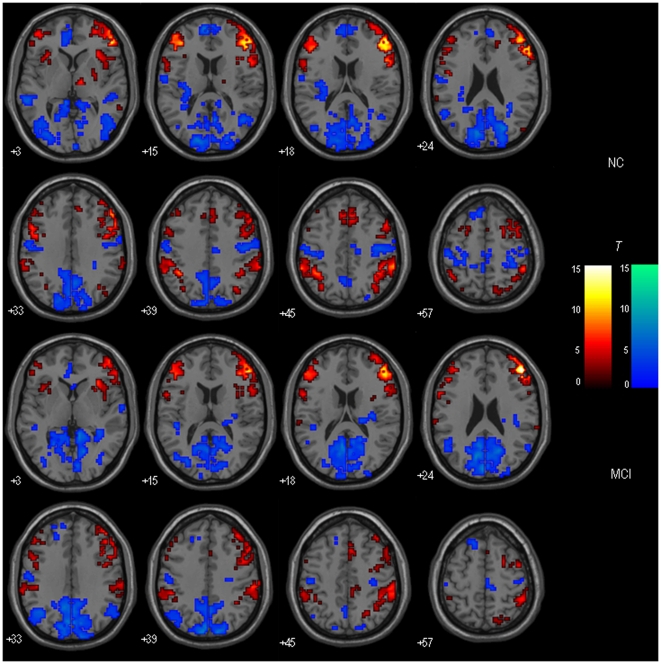
Within-group maps of the right DLPFC-FC in MCI and healthy control groups. The voxels with hot color represent DLPFC positive functional connectivity, and the voxels with cold color represent DLPFC negative functional connectivity. The statistical threshold was set at P<0.01 and cluster size > = 405 mm^3^, which corresponded to a corrected P<0.05. Left is the left.

Figure 1 and Figure 2 respectively show the bilateral DLPFC-FC maps for MCI patients and healthy controls. Visual inspection indicated that the two groups showed the similar patterns of functional connectivity in the bilateral DLPFC. The DLPFC showed strong functional connectivity to a distributed regions including middle frontal gyrus (BA 6, 11, 46, 47), medial frontal gyrus (BA 6, 8), inferior parietal lobule (IPL; BA 40), insula (BA 13), anterior cingulate cortex (ACC; BA 24/32), thalamus, putamen/caudate, etc.

**Figure 3:**
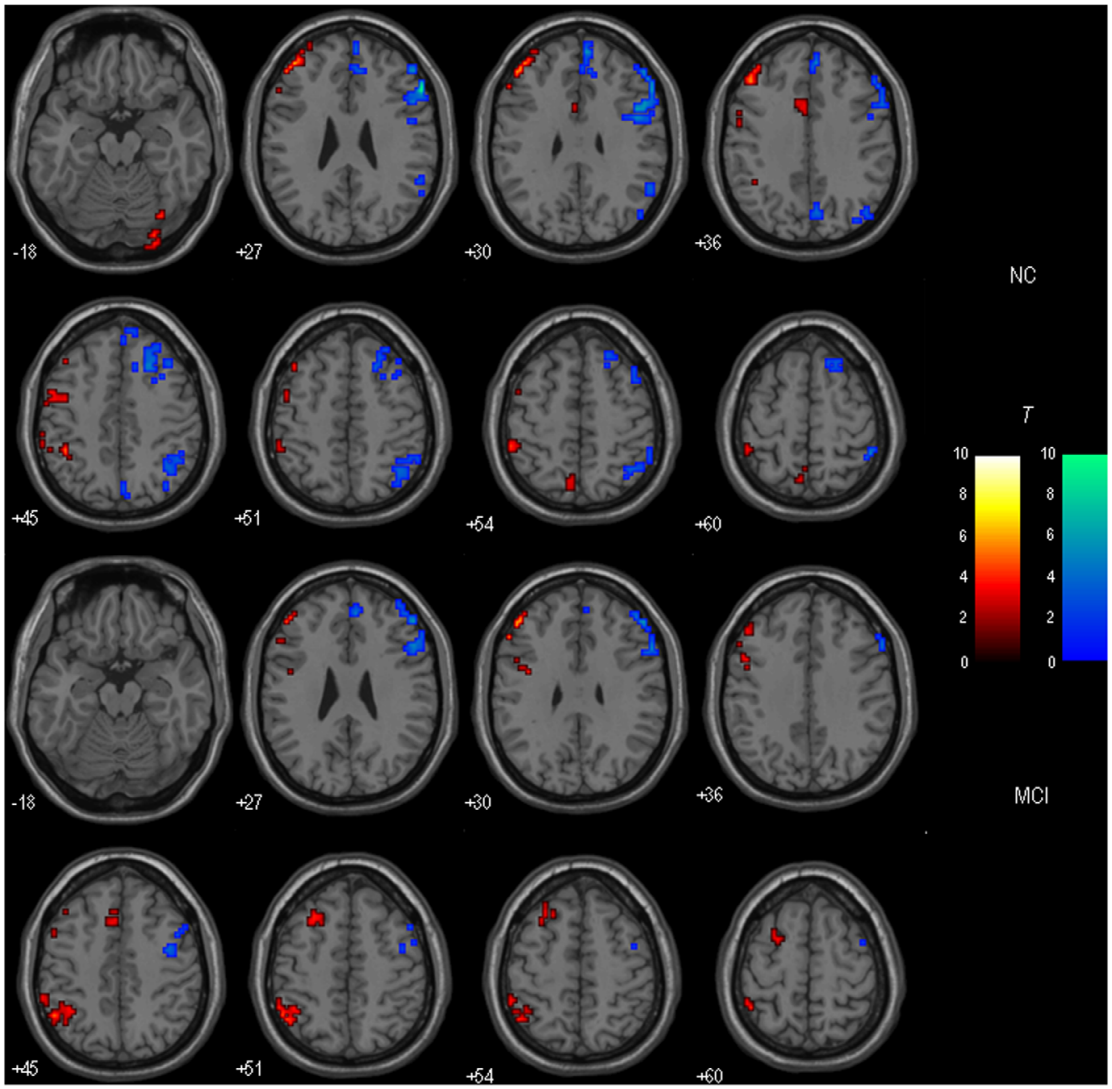
DLPFC-FC asymmetry of Left vs. Right (hot color) and Right vs. Left (cold color) in healthy controls (Up), and DLPFC-FC asymmetry of Left vs. Right (hot color) and Right vs. Left (cold color) in MCI patients (Down). The threshold was set at *p*<0.01 with cluster size > = 216 mm^3^, which corresponded to a corrected threshold of *p*<0.05. Left is the left.

The left DLPFC-FC and the right DLPFC-FC were statistically contrasted for healthy controls and MCI patients respectively. Figure 3 showed the results of within-group DLPFC-FC asymmetry.

### DLPFC-FC: Between-group Results

**Figure 4:**
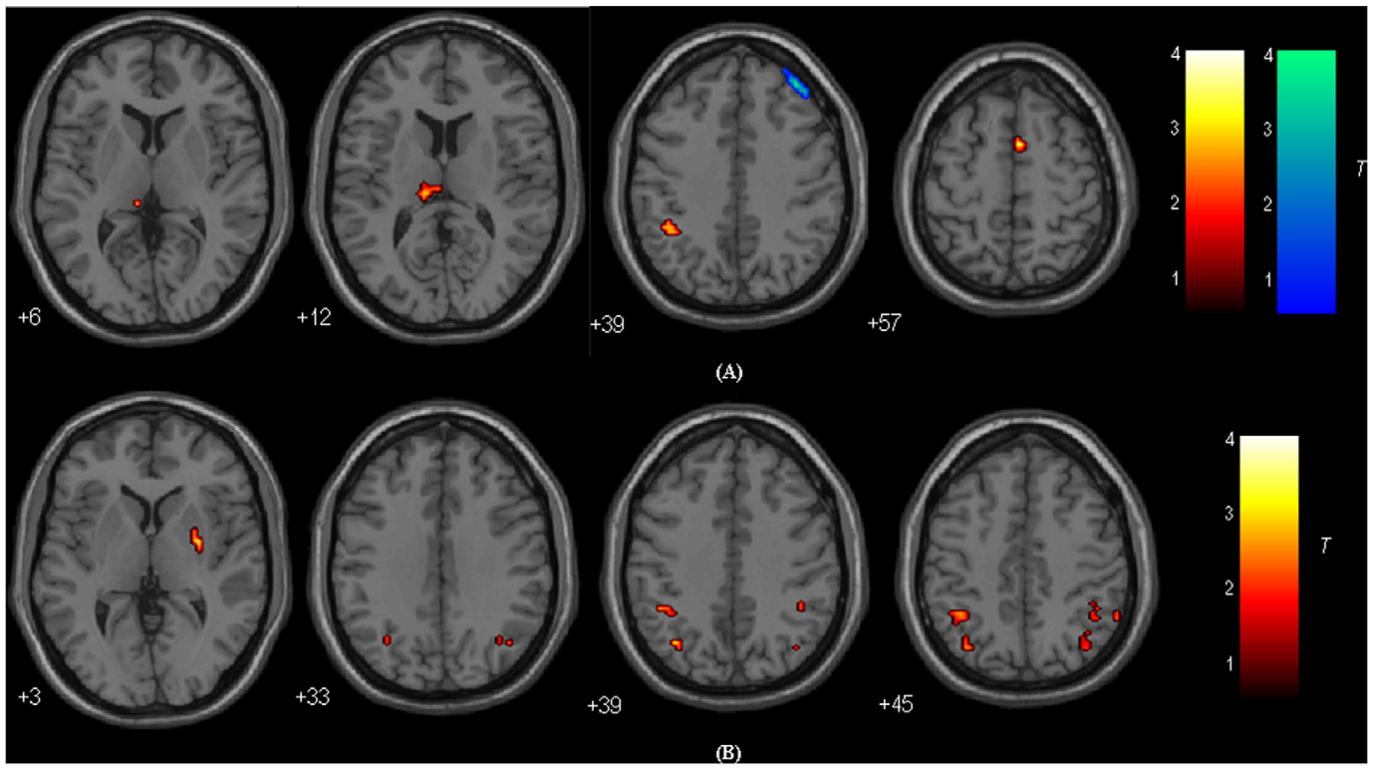
Regions showing significant differences in functional connectivity to the left DLPFC (Up) and right DLPFC (Down) between MCI patients and healthy controls (with GM correction). For the details of the regions, see Table 4 and Table 5. Areas in hot color indicate brain regions with significantly decreased functional connectivity with the left/right DLPFC in MCI patients compared to healthy controls. Areas in cold color indicate brain regions with significantly increased functional connectivity with the left/right DLPFC in MCI patients compared to healthy controls. The threshold was set at *p*<0.01 with cluster size > = 216 mm^3^, which corresponded to a corrected threshold of *p*<0.05. Of note, we showed the two-sample t-test results were attained within a corresponding mask by combining the one sample t-test maps of the two groups (Figure 1 and Figure 2). Left is the left.

Figure 4(A) and Table 2 show the regions with group differences between MCI patients and healthy controls for the left DLPFC-FC. Comparing with healthy controls, decreased functional connectivity with the left DLPFC was observed in MCI patients in the right superior frontal gyrus (BA 6), left inferior/superior parietal lobule (IPL/SPL; BA 39/7, 40), and left thalamus, while increased functional connectivity with the left DLPFC was observed in MCI patients in the right middle frontal gyrus (BA 9).

**Table 2:** Regions showing the group differences in the left DLPFC-FC (without GM correction).

Regions	BA	Cluster	Coordinates (MNI)			T-score
**NC vs. MCI**						
Rt. superior frontal gyrus	6	26	3	6	57	3.40
Lt. inferior parietal lobule	39	16	−36	−66	39	3.06
Lt. superior parietal lobule	7		−33	−75	45	1.79
Lt. inferior parietal lobule	40	18	−39	−48	39	3.05
Lt. thalamus		18	−12	−24	12	3.58
			−9	−30	6	3.26
**MCI vs. NC**						
Rt. middle frontal gyrus	9	25	36	42	39	3.13
			33	54	36	3.09

DLPFC-FC, dosolateral prefrontal cortex functional connectivity; BA, Brodmann's area; Lt., left; Rt., right; P<0.05, corrected for multiple comparison.

Figure 4(B) and Table 3 show the regions with the right DLPFC-FC differences between MCI patients and healthy controls. Comparing with healthy controls, decreased functional connectivity with the right DLPFC was observed in MCI patients in the left middle/inferior frontal gyrus (BA 11/47), right middle frontal gyrus (BA 6), right medial frontal gyrus (BA 9), left IPL/SPL (BA 39/7), left supramarginal gyrus (BA 40), right IPL/SPL/angular gyrus (BA 39, 7, 40), and right putamen, while no regions showed increased connectivity with the right DLPFC in MCI patients.

**Table 3:** Regions showing the group differences in the right DLPFC-FC (without GM correction).

Regions	BA	Cluster	Coordinates (MNI)			T-score
**NC vs. MCI**						
Lt. middle frontal gyrus	11	16	−27	42	−12	2.82
Lt. inferior frontal gyrus	47		−27	33	−12	2.60
Rt. middle frontal gyrus	6	16	42	9	60	3.06
Rt. medial frontal gyrus	9	23	6	36	36	2.60
Lt. inferior parietal lobule	39	29	−36	−66	39	3.77
Lt. superior parietal lobule	7		−39	−63	51	2.69
Lt. supramarginal gyrus	40	89	−60	−48	36	2.96
Lt. inferior parietal lobule			−39	−48	45	2.87
Rt. inferior parietal lobule	39	30	39	−69	42	2.80
Rt. angular gyrus			42	−66	33	2.63
Rt. superior parietal lobule	7		39	−63	51	2.12
Rt. inferior parietal lobule	40	58	42	−45	42	2.66
			57	−51	45	2.63
Rt. putamen		21	30	−3	3	4.04
			30	−6	−9	3.71
**MCI vs. NC**						
None						

DLPFC-FC, dosolateral prefrontal cortex functional connectivity; BA, Brodmann's area; Lt., left; Rt., right; P<0.05, corrected for multiple comparison.

**Figure 5:**
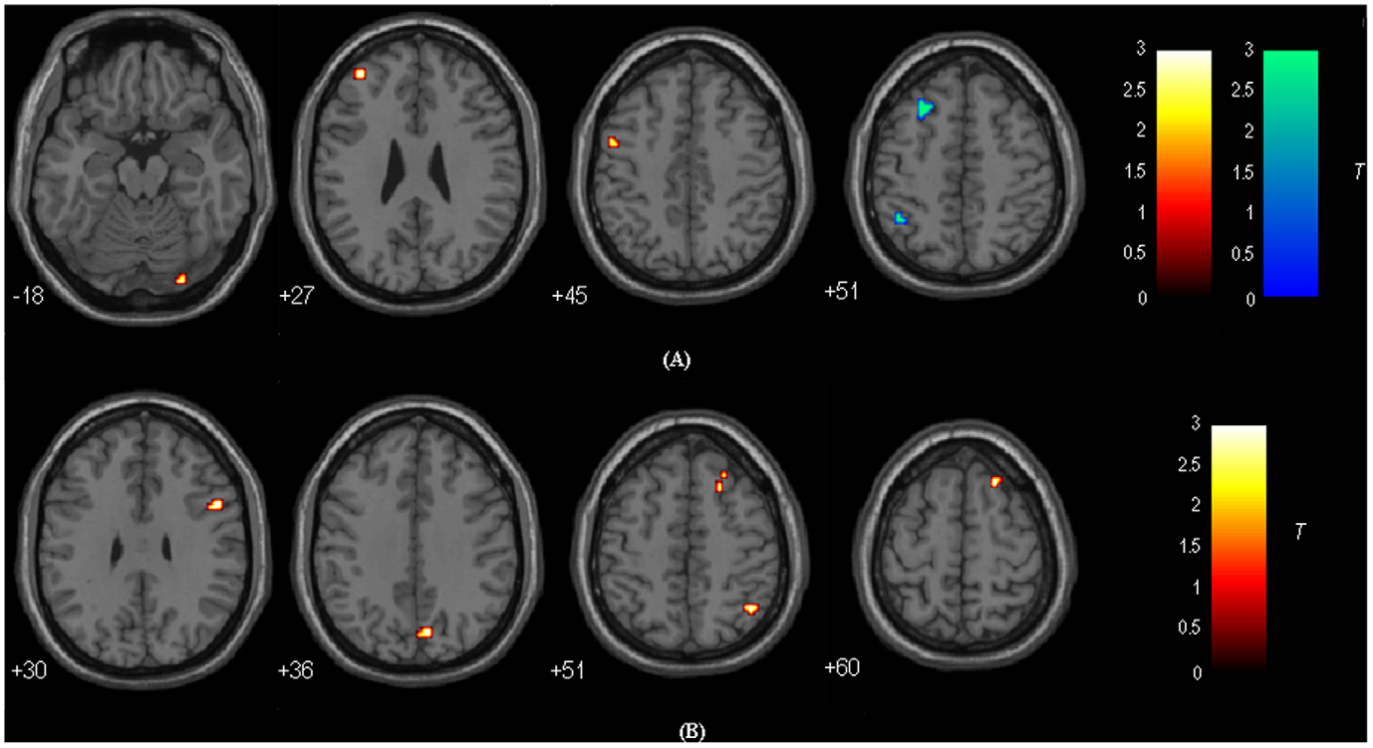
Regions showing significant group differences in the left lateralized (Up) and right lateralized (Down) DLPFC-FC asymmetry. Areas in hot color indicate significant decreased lateralized DLPFC-FC asymmetry, and areas in cold color indicate significant increased lateralized DLPFC-FC asymmetry in MCI patients compared to healthy controls. The threshold was set at p<0.01 with cluster size > = 162 mm^3^, which corresponded to a corrected threshold of p<0.05. Left is the left.

Figure 5 shows the regions with the DLPFC-FC asymmetry differences between MCI patients and healthy controls. As contrast to healthy controls, MCI patients showed decreased left lateralized DLPFC-FC in the left superior frontal gyrus (BA 10), middle frontal gyrus (BA 6) and right inferior occipital gyrus (BA 18), while decreased right lateralized DLPFC-FC in the right IPL (BA 40), PCu (BA 7), superior frontal gyrus (BA 6, 8) and middle frontal gyrus (BA 9). In addition, compared with healthy controls, MCI patients also showed increased left lateralized DLPFC-FC in the left middle frontal gyrus (BA 8) and IPL (BA 40).

Additionally, Figures S1, S2, S3 and S4 in the supplementary material show the within-group results of the coordinate-based DLPFC-FC, and Figures S7 and S8 show the between-group results. The DLPFC-FC patterns are similar to those using BA 46 as DLPFC seeds. Figures S5 and S6 show the within-group results of PCC-FC, and Figure S9 show the group differences. As contrast to healthy controls, the connectivities between PCC and most of DMN regions, including PCC, angular gurus, medial frontal gyrus and superior/middle frontal gyrus, were significantly reduced, which are consistent with previous studies [17], [18].

### Correlations between DLPFC-FC and Neuropsychological Measures

**Figure 6:**
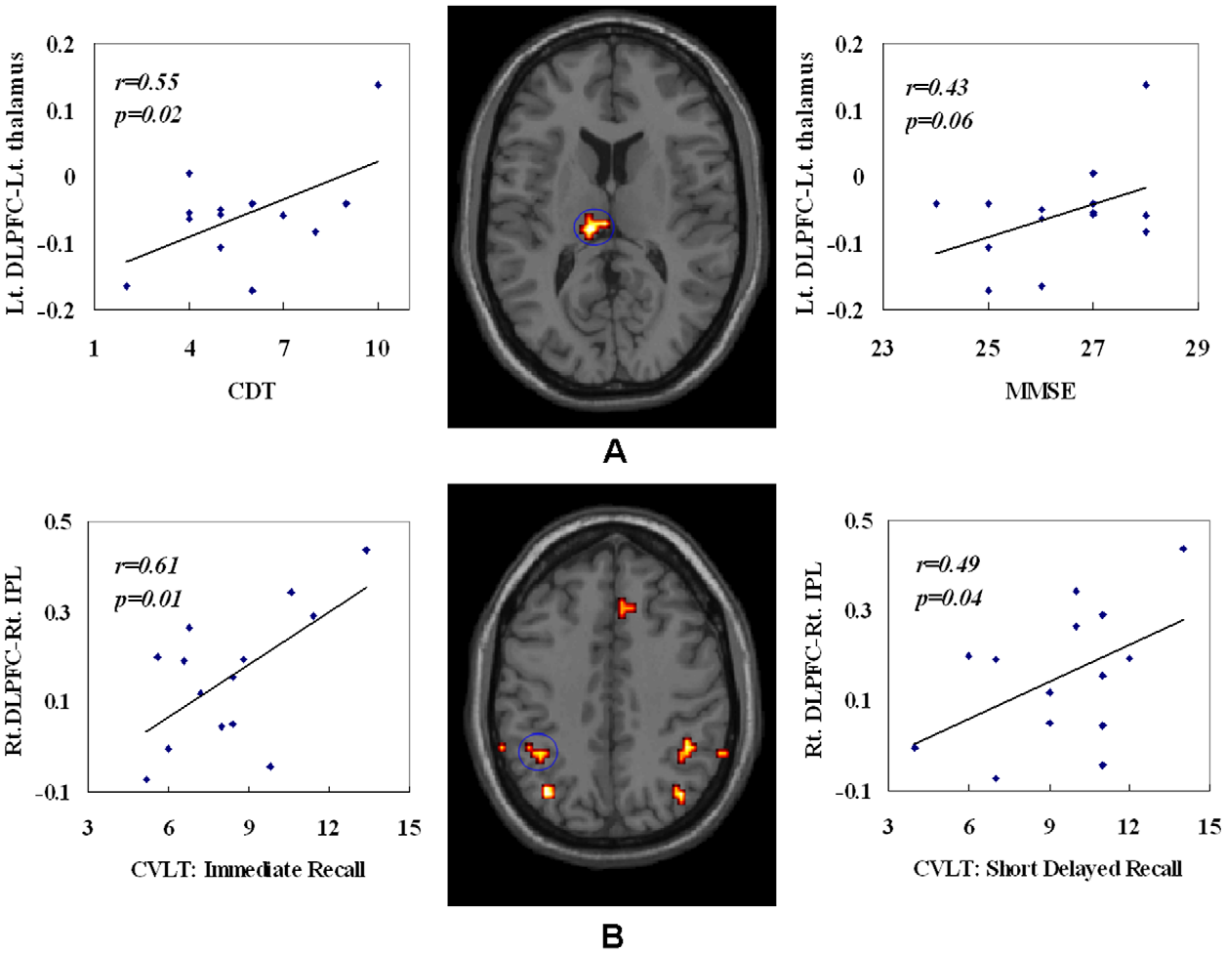
Scatter plots of the association in MCI group between the connectivity of the left DLPFC with a cluster in the left thalamus (A), the connectivity of the right DLPFC with a cluster in the right IPL (B) and the neuropsychological scores (including MMSE, CDT, CVLT) (without GM correction). The two connections were found to be significantly decreased in MCI patients as compared to healthy controls.

As shown in Figure 6 (A) and (B), correlation analysis revealed that two clusters showed significant correlation between DLPFC-FC and neuropsychological performances in MCI patients. The connectivity of the left DLPFC with a cluster in the left thalamus showed significant or near significant correlation with CDT (r = 0.55, *P* = 0.02) and MMSE (r = 0.43, *P* = 0.06). The connectivity between the right DLPFC and a cluster in the right IPL was significantly correlated with CVLT (Immediate Recall) (r = 0.61, *P* = 0.01) and CVLT (Short Delayed Recall) (r = 0.49, *P* = 0.04). All the other correlations were not significant (*P*>0.1). The post hoc multiple comparisons of the correlations between DLPFC-FC and neuropsychological measures were also done to correct for the number of neuropsychological measures (See Supplementary Materials: 2 for more details).

### DLPFC-FC: Controlling for Regional GM Atrophy

A VBM analysis was performed to reveal the GM volume differences between MCI patients and healthy controls. The results showed most significant atrophies in the frontal, temporal, parietal, occipital and sub-cortical regions (See Figure S10 (A) and (B) in Supplementary Materials), and the global maxima atrophy was located in the precuneus. This is consistent with numerous previous studies of MCI [45], [46] and AD patients [44], [47].

By taking into account the GM atrophy as a covariate, we found that statistical significance was reduced in the regions showed decreased DLPFC-FC in MCI patients as compared to healthy controls (as shown in Table 4 and Table 5). For example, there was a 26 voxels' cluster (*T* = 3.40) in the right superior frontal gyrus (BA 6) showing significantly group differences (Table 2) but the cluster decreased to 22 voxels (*T* = 3.38) after the GM correction (Table 4). However, the region in the right middle frontal gyrus (Table 2, 4) which showed increased DLPFC-FC in MCI patients as compared to healthy controls were not affected (The maximal *T* value changed from 3.13 to 3.27, although the voxel number changed from 25 to 24.).

**Table 4:** Regions showing the group differences in the left DLPFC-FC (with GM correction).

Regions	BA	Cluster	Coordinates (MNI)			T-score
**NC vs. MCI**						
Rt. superior frontal gyrus	6	22	3	6	57	3.38
Lt. supramarginal gyrus/inferior parietal lobule	40	16	−45	−45	36	2.66
Lt. thalamus		16	−9	−30	6	3.52
			−12	−24	12	3.06
**MCI vs. NC**						
Rt. middle frontal gyrus	9	24	36	42	39	3.27

DLPFC-FC, dosolateral prefrontal cortex functional connectivity; BA, Brodmann's area; Lt., left; Rt., right; P<0.05, corrected for multiple comparison.

**Table 5:** Regions showing the group differences in the right DLPFC-FC (with GM correction).

Regions	BA	Cluster	Coordinates (MNI)			T-score
**NC vs. MCI**						
Lt. inferior parietal lobule	39	24	−36	−66	39	3.14
Lt. superior parietal lobule	7		−39	−63	51	2.73
Lt. supramarginal gyrus	40	44	−45	−45	36	2.97
Lt. inferior parietal lobule			−39	−48	45	2.64
Rt. angular gyrus	39	20	42	−66	33	2.50
Rt. Inferior parietal lobule			39	−69	42	2.35
Rt. inferior parietal lobule	40	31	57	−51	45	2.43
			42	−45	42	2.37
Rt. putamen		20	30	−3	3	3.43
			30	−6	−9	3.10
**MCI vs. NC**						
None						

DLPFC-FC, dosolateral prefrontal cortex functional connectivity; BA, Brodmann's area; Lt., left; Rt., right; P<0.05, corrected for multiple comparison.

When controlling for the GM atrophy, the correlations between DLPFC-FC and neuropsychological performances were also changed. While the correlation trend were still reserved, the significant correlations between the connectivity of the left DLPFC with the left thalamus and CDT (r = 0.38, *P* = 0.1), MMSE (r = 0.20, *P* = 0.26) were reduced to be not significant. The significant correlations between the connectivity of the right DLPFC with the right IPL and CVLT (Immediate Recall) (r = 0.64, *P* = 0.01), CVLT (Short Delayed Recall) (r = 0.48, *P* = 0.05) were kept.

## Discussion

The present study utilized bilateral DLPFC as seeds to compare the DLPFC-FC differences between MCI patients and healthy controls. The DLPFC-FC patterns of healthy controls in the current study covered the two pathways: fronto-parietal circuits and fronto-striatial-thalamus circuits. This is consistent with a previous study [27]. The fronto-parietal and fronto-striatial-thalamus circuits in the current study appear to share the majority elements of the proposed fronto-parietal network and cingulo-opercular network in Dosenbach et al. [27]. Using DLPFC (BA 46) as seeds enable us to follow the two pathways simultaneously, while only parts of them were traced using other regions as seeds [31]. The DLPFC-FC pattern of healthy controls acting as a baseline, the effects of functional disconnection and compensation together with the altered asymmetry were observed in MCI patients.

### Functional Disconnection in MCI Patients

#### Fronto-parietal Disconnection

Comparing to healthy elderly, MCI patients showed the significant reduced parietal connectivity with the DLPFC. This disconnection effect was still strong even after controlling for GM atrophy. These results demonstrated a functional disconnection within a distributed frontal-parietal network, and confirmed that disconnection between the anterior and the posterior regions can also be detected in MCI, the early stage of AD.

Moreover, significant correlations between the right DLPFC-FC with a cluster in the right IPL and two CVLT scores (i.e., Immediate Recall and Short Delayed Recall) were observed.

When controlling for GM atrophy, the two correlations were still significant. This may suggest that this disconnection contributes to the impairment of episodic memory retrieval in MCI patients, which was measured using CVLT. Previous findings demonstrated that the right DLPFC and parietal cortex were recruited in episodic retrieval, while the left prefrontal cortex was associated with episodic encoding [48], which may give explanations to the effect of the right lateralization.

#### Fronto-striatial-thalamus Disconnection

We found a region in the medial/superior frontal cortex and some sub-cortical regions including thalamus and putamen showed decreased connectivity with the DLPFC even controlling for GM atrophy. This finding extends the previous studies and implies that the fronto-striatial-thalamus connection may also be disrupted in MCI patients.

Without GM atrophy correction, the correlation between the connectivity of the left DLPFC with a cluster in the left thalamus and CDT and MMSE was significant. After controlling for GM atrophy, the correlation is still positive while the statistic power is reduced. This finding implies the existed fronto-striatial-thalamus disconnection can partially explain the executive function decline (measured by CDT) and disease severity (measured by MMSE) in MCI patients, while GM atrophy may also in part contribute to these effects.

Taking together, based on the facts that the fronto-parietal and fronto-striatial-thalamus circuits are highly correlated with human cognition, our findings suggested that cognitive impairments in MCI may be related to a reduction in the integrated activity of these circuits.

### Within-frontal Functional Compensation in MCI Patients

An increased right frontal connectivity with the left DLPFC was also observed in this study.

Previous studies have reported increased activity in the right DLPFC [49], [50], and increased functional connectivity between the right prefrontal cortex and other regions [17] in MCI patients using resting state or various cognitive tasks. This study is also consistent with a recent task-fMRI study of our group [51], in which number series completion task was used to examine the inductive reasoning ability of MCI patients. MCI patients and healthy controls showed similar accuracies, reaction times and the activation pattern of the bilateral DLPFC. However, the functional connectivity between the left and right DLPFC in MCI patients was significantly stronger than that in healthy controls (as shown in Figure S11 in Supplementary Materials). Consistent with previous studies, the current finding may suggest a compensation mechanism of right prefrontal cortex in MCI patients. It was thus postulated that MCI patients may use additional neural resources in the right prefrontal regions to compensate for losses of cognitive functions.

### Altered DLPFC-FC asymmetry in MCI patients

It was found that, as compared to the healthy controls, the DLPFC-FC asymmetries in MCI patients were altered (see Figure 5). The current study then extends the previous findings of brain regional asymmetry (either structural or functional) in MCI patients [52], [53] to the domain of resting state functional connectivity. Brain functional asymmetry is a fundamental characteristic of the intrinsic functional architecture of the brain [54], and the asymmetry phenomena and its developmental trajectory in the whole life-span have been observed using resting-state fMRI [55]. Thus, the altered functional asymmetry in MCI may underlie its physiological mechanism, and complement for the above findings of DLPFC-FC functional disconnection and compensation.

### Comparison with a Previous Study

DLPFC-FC has also been used in first-episode schizophrenia (FES) patients. Zhou et al. [41] also used bilateral BA46 as seeds and examined the functional dysconnectivity of the DLPFC in FES based on resting-state fMRI. They found that the bilateral DLPFC showed reduced functional connectivity to the parietal lobe, posterior cingulate cortex (PCC), thalamus and striatum and enhanced functional connectivity between the left DLPFC and the left mid-posterior temporal lobe and the paralimbic regions in FES patients. The similar functional dysconnectivity associated with the DLPFC exists both in FES and MCI patients, however, the increased connectivites are different. Considering the potential confounding factors involved in the two group patients (e.g., age, gender, scanning protocols, experimenter, etc.), whether the functional disconnection and functional compensation in this study are specific for MCI is still unclear, and further comparative studies should be performed in the future.

### Limitations and Further Considerations

There are still technical and biological limitations in the current study. The first limitation of our present study lies in the effects of physiological noises such as cardiac and respiratory rhythms. We used a relatively low sampling rate (TR = 2 s) for multi-slice acquisitions. According to the Nyquist sampling theorem, high frequency noises such as cardiac rhythm can not be completely removed just by band-pass filtering of 0.01–0.08 Hz. In the future, these physiological effects may be estimated and removed by simultaneously recording the respiratory and cardiac cycle during the data acquisition. The second limitation is that we cannot exclude the possibility that the group differences of DLPFC-FC, to some extent, resulted from different spontaneous thoughts between the two groups, given that the resting state is associated with spontaneous thoughts and internal cognitive processing. In the future, on the one hand, a large number of samples will be collected. By considering spontaneous thoughts as events of no interest, “grand average” on big samples may be statistically helpful. On the other hand, we will ask subjects to report their spontaneous thoughts after scanning, which may contribute to exploring the behavioral group differences in such a resting state study. Additionally, this study was cross-sectional. It has been suggested that about 1/3 of MCI patients in this study develop AD. It would be important in the future to explore changes in DLPFC-FC between those who do and do not progress to AD, and to investigate longitudinal changes in MCI/AD patients.

In summary, abnormal functional connectivity of the DLPFC in MCI patients was for the first time examined in the present study. The findings demonstrated the coexistence of the fronto-parietal and fronto-striatial-thalamus disconnections and within-prefrontal-lobe compensation in MCI patients.

## Supporting Information

Figure S1 Within-group maps of the left DLPFC-FC in healthy control groups. The voxels with hot color represent DLPFC positive functional connectivity, and the voxels with cold color represent DLPFC negative functional connectivity. The statistical threshold was set at a corrected P<0.05. Left is the left.
(TIF)

Figure S2 Within-group maps of the left DLPFC-FC in MCI groups. The voxels with hot color represent DLPFC positive functional connectivity, and the voxels with cold color represent DLPFC negative functional connectivity. The statistical threshold was set at a corrected P<0.05. Left is the left.
(TIF)

Figure S3 Within-group maps of the right DLPFC-FC in healthy control groups. The voxels with hot color represent DLPFC positive functional connectivity, and the voxels with cold color represent DLPFC negative functional connectivity. The statistical threshold was set at a corrected P<0.05. Left is the left.
(TIF)

Figure S4 Within-group maps of the right DLPFC-FC in MCI groups. The voxels with hot color represent DLPFC positive functional connectivity, and the voxels with cold color represent DLPFC negative functional connectivity. The statistical threshold was set at a corrected P<0.05. Left is the left.
(TIF)

Figure S5 Within-group maps of the PCC-FC in NC groups. The voxels with hot color represent PCC positive functional connectivity, and the voxels with cold color represent PCC negative functional connectivity. The statistical threshold was set at a corrected P<0.05. Left is the left.
(TIF)

Figure S6 Within-group maps of the PCC-FC in MCI groups. The voxels with hot color represent PCC positive functional connectivity, and the voxels with cold color represent PCC negative functional connectivity. The statistical threshold was set at a corrected P<0.05. Left is the left.
(TIF)

Figure S7 Regions showing significant group differences in functional connectivity to the left DLPFC. Areas in hot color indicate brain regions with significantly decreased functional connectivity with the left DLPFC in MCI patients compared to healthy controls. Areas in cold color indicate brain regions with significantly increased functional connectivity with the left DLPFC in MCI patients compared to healthy controls. The threshold was set at a corrected threshold of p<0.05. Left is the left.
(TIF)

Figure S8 Regions showing significant group differences in functional connectivity to the right DLPFC. Areas in hot color indicate brain regions with significantly decreased functional connectivity with the right DLPFC in MCI patients compared to healthy controls. No regions showed significantly increased functional connectivity with the right DLPFC in MCI patients compared to healthy controls. The threshold was set at a corrected threshold of p<0.05. Left is the left.
(TIF)

Figure S9 Regions showing significant group differences in PCC functional connectivity. Areas in hot color indicate brain regions with significantly decreased PCC functional connectivity in MCI patients compared to healthy controls. No regions showed significantly increased PCC functional connectivity in MCI patients compared to healthy controls. The threshold was set at a corrected threshold of p<0.05. Left is the left.
(TIF)

Figure S10 Axial view (A) and sagittal view (B) of the T-statistical maps of GM volume differences between MCI and healthy controlling using a voxel-based-mophometry method. There were significant differences in the frontal, temporal, parietal, occipital lobe and sub-cortical regions. The statistical threshold was set at P<0.001 and cluster size >324 mm3, which corresponded to a corrected P<0.05. Left is the left.
(TIF)

Figure S11 The BOLD response in the bilateral DLPFC region during number series completion task. The connectivity between the bilateral DLPFC in MCI patients was stronger than that of normal controls. This figure was amended from Yang et al. (2009).
(TIF)
