# Supplementary figures and images for: Functional Disconnection and Compensation in Mild Cognitive Impairment: Evidence from DLPFC Connectivity Using Resting-State fMRI
Source: PLoS One. 2011 Jul 21;6(7):e22153. doi: 10.1371/journal.pone.0022153 (PMC3141010; doi:10.1371/journal.pone.0022153)

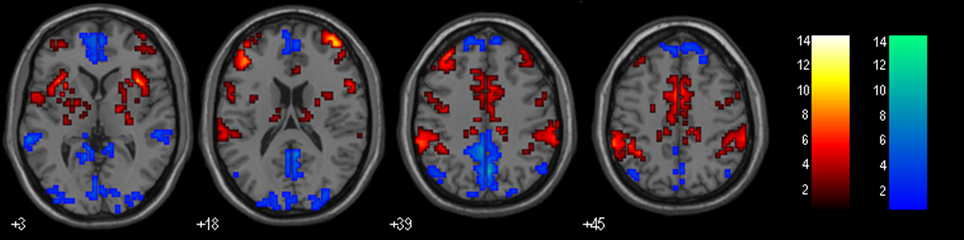

Supplement: Figure S1 — Within-group maps of the left DLPFC-FC in healthy control groups. The voxels with hot color represent DLPFC positive functional connectivity, and the voxels with cold color represent DLPFC negative functional connectivity. The statistical threshold was set at a corrected P<0.05. Left is the left. (TIF) [file pone.0022153.s001.tif]

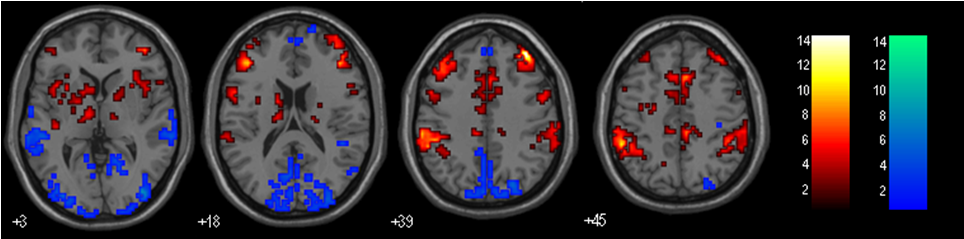

Supplement: Figure S2 — Within-group maps of the left DLPFC-FC in MCI groups. The voxels with hot color represent DLPFC positive functional connectivity, and the voxels with cold color represent DLPFC negative functional connectivity. The statistical threshold was set at a corrected P<0.05. Left is the left. (TIF) [file pone.0022153.s002.tif]

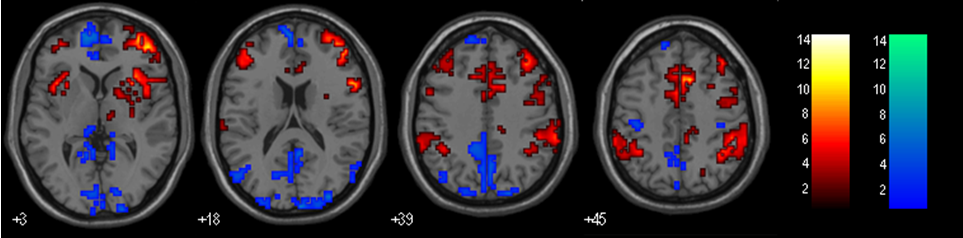

Supplement: Figure S3 — Within-group maps of the right DLPFC-FC in healthy control groups. The voxels with hot color represent DLPFC positive functional connectivity, and the voxels with cold color represent DLPFC negative functional connectivity. The statistical threshold was set at a corrected P<0.05. Left is the left. (TIF) [file pone.0022153.s003.tif]

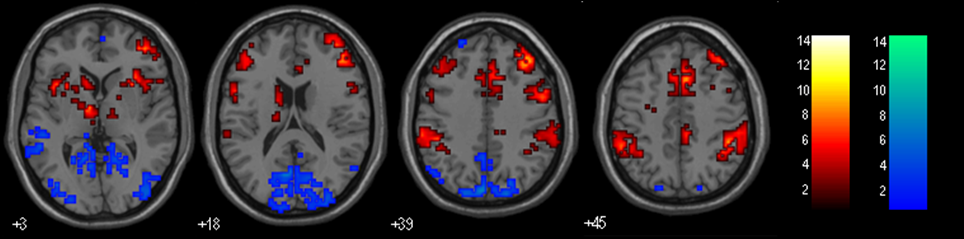

Supplement: Figure S4 — Within-group maps of the right DLPFC-FC in MCI groups. The voxels with hot color represent DLPFC positive functional connectivity, and the voxels with cold color represent DLPFC negative functional connectivity. The statistical threshold was set at a corrected P<0.05. Left is the left. (TIF) [file pone.0022153.s004.tif]

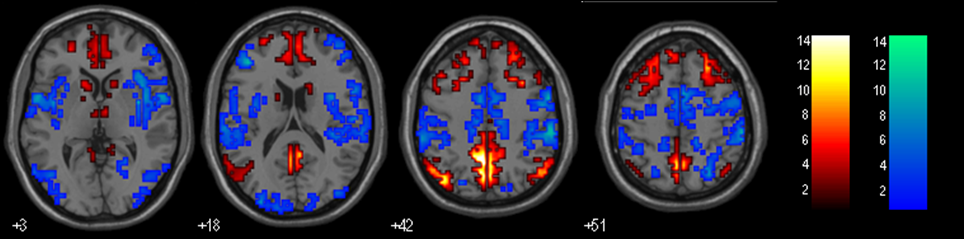

Supplement: Figure S5 — Within-group maps of the PCC-FC in NC groups. The voxels with hot color represent PCC positive functional connectivity, and the voxels with cold color represent PCC negative functional connectivity. The statistical threshold was set at a corrected P<0.05. Left is the left. (TIF) [file pone.0022153.s005.tif]

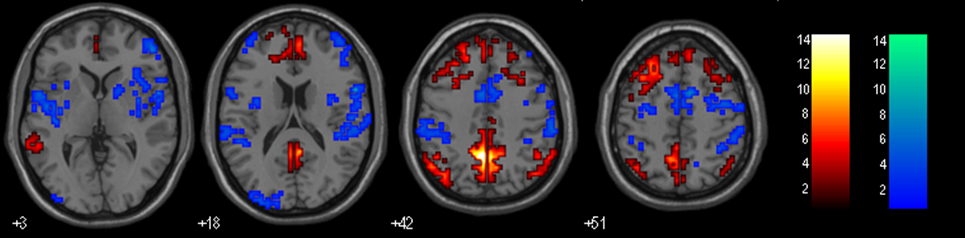

Supplement: Figure S6 — Within-group maps of the PCC-FC in MCI groups. The voxels with hot color represent PCC positive functional connectivity, and the voxels with cold color represent PCC negative functional connectivity. The statistical threshold was set at a corrected P<0.05. Left is the left. (TIF) [file pone.0022153.s006.tif]

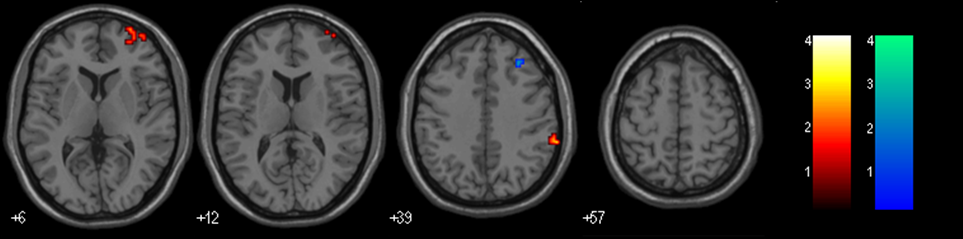

Supplement: Figure S7 — Regions showing significant group differences in functional connectivity to the left DLPFC. Areas in hot color indicate brain regions with significantly decreased functional connectivity with the left DLPFC in MCI patients compared to healthy controls. Areas in cold color indicate brain regions with significantly increased functional connectivity with the left DLPFC in MCI patients compared to healthy controls. The threshold was set at a corrected threshold of p<0.05. Left is the left. (TIF) [file pone.0022153.s007.tif]

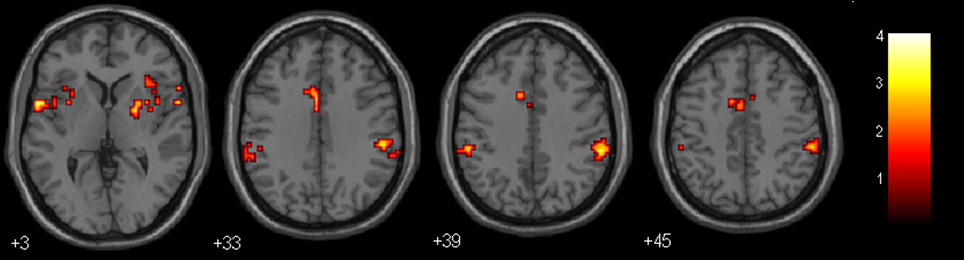

Supplement: Figure S8 — Regions showing significant group differences in functional connectivity to the right DLPFC. Areas in hot color indicate brain regions with significantly decreased functional connectivity with the right DLPFC in MCI patients compared to healthy controls. No regions showed significantly increased functional connectivity with the right DLPFC in MCI patients compared to healthy controls. The threshold was set at a corrected threshold of p<0.05. Left is the left. (TIF) [file pone.0022153.s008.tif]

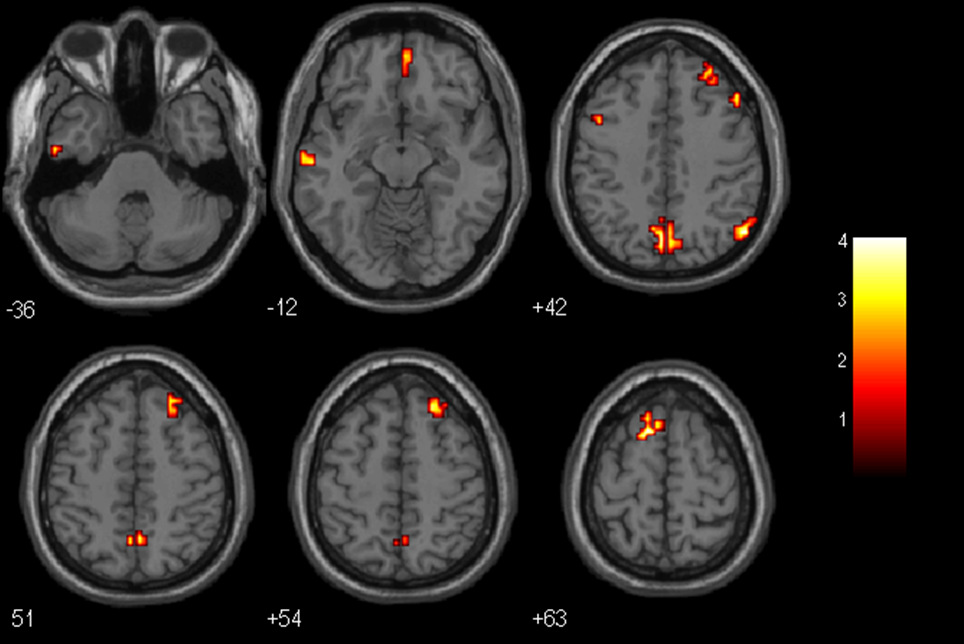

Supplement: Figure S9 — Regions showing significant group differences in PCC functional connectivity. Areas in hot color indicate brain regions with significantly decreased PCC functional connectivity in MCI patients compared to healthy controls. No regions showed significantly increased PCC functional connectivity in MCI patients compared to healthy controls. The threshold was set at a corrected threshold of p<0.05. Left is the left. (TIF) [file pone.0022153.s009.tif]

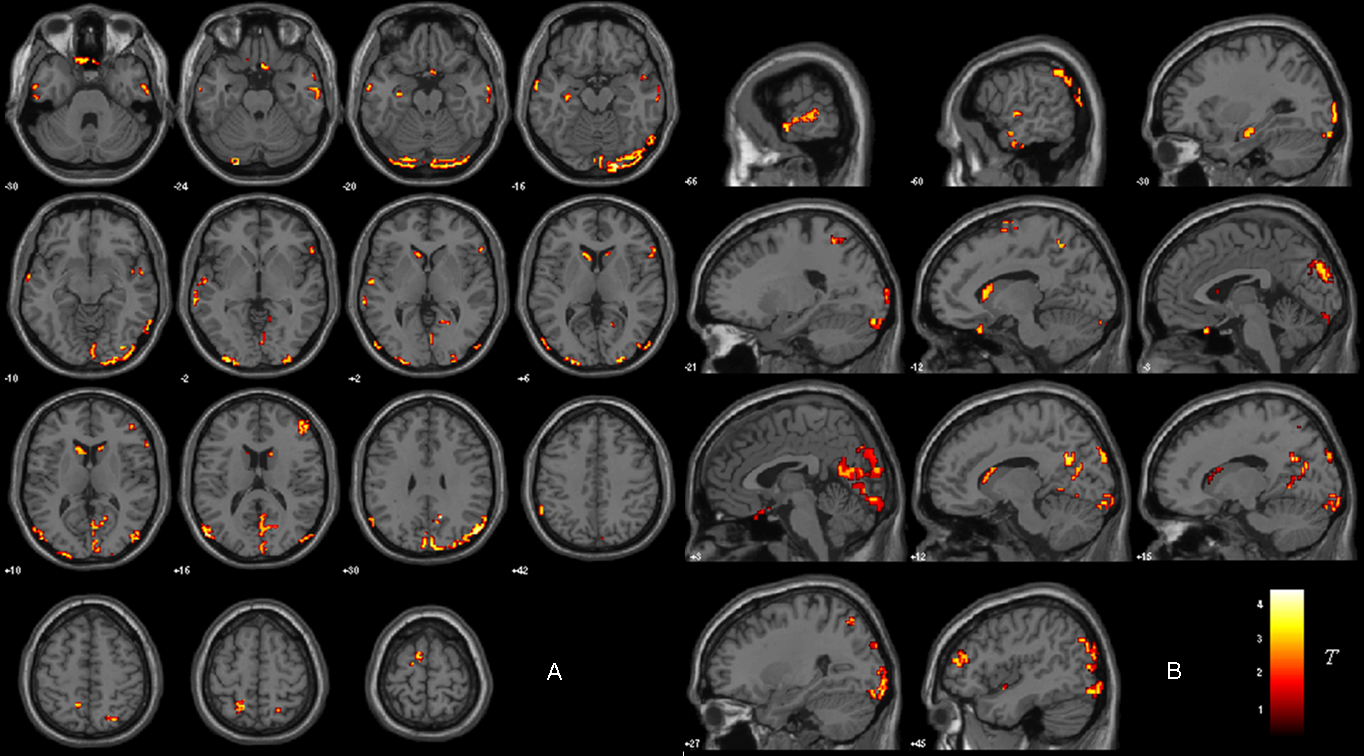

Supplement: Figure S10 — Axial view (A) and sagittal view (B) of the T-statistical maps of GM volume differences between MCI and healthy controlling using a voxel-based-mophometry method. There were significant differences in the frontal, temporal, parietal, occipital lobe and sub-cortical regions. The statistical threshold was set at P<0.001 and cluster size >324 mm3, which corresponded to a corrected P<0.05. Left is the left. (TIF) [file pone.0022153.s010.tif]

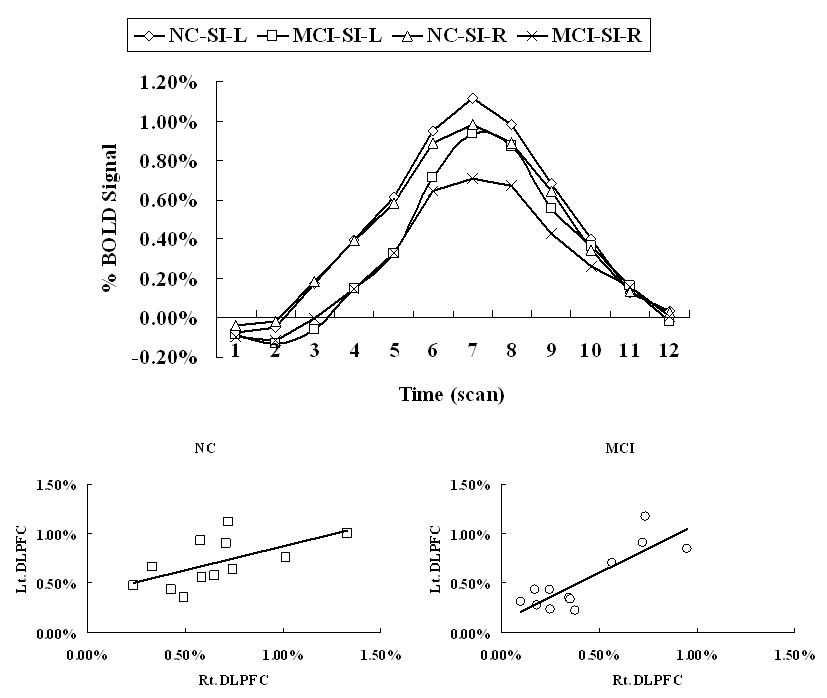

Supplement: Figure S11 — The BOLD response in the bilateral DLPFC region during number series completion task. The connectivity between the bilateral DLPFC in MCI patients was stronger than that of normal controls. This figure was amended from Yang et al. (2009). (TIF) [file pone.0022153.s011.tif]
